# Aortic annular plane systolic excursion in cats with hypertrophic cardiomyopathy

**DOI:** 10.1111/jvim.16962

**Published:** 2023-12-01

**Authors:** Luke C. Dutton, Ilaria Spalla, Joonbum Seo, Joel Silva, Jose Novo Matos

**Affiliations:** ^1^ Department of Veterinary Medicine University of Cambridge Cambridge UK; ^2^ Ospedale Veterinario San Francesco Milan Italy; ^3^ Animal Referral Centre Auckland New Zealand; ^4^ North Downs Specialist Referrals, The Friesian Buildings 3 & 4 Bletchingley, Surrey UK

**Keywords:** echocardiography, feline, mitral annular plane systolic excursion, strain, systolic function

## Abstract

**Background:**

Impairment of left ventricular (LV) longitudinal function is an early marker of systolic dysfunction in hypertrophic cardiomyopathy (HCM). Aortic annular plane systolic excursion (AAPSE) is a measure of LV longitudinal function in people that has not been evaluated in cats.

**Hypothesis:**

Aortic annular plane systolic excursion is lower in cats with HCM compared to control cats, and cats in stage C have the lowest AAPSE.

**Animals:**

One hundred seventy‐five cats: 60 normal, 61 HCM stage B and 54 HCM stage C cats.

**Materials:**

Multicenter retrospective case‐control study. Electronic medical records from 4 referral hospitals were reviewed for cats diagnosed with HCM and normal cats. HCM was defined as LV wall thickness ≥6 mm and normal cats ≤5 mm. M‐mode bisecting the aorta in right parasternal short‐axis view was used to measure AAPSE.

**Results:**

Aortic annular plane systolic excursion was lower in HCM cats compared to normal cats (3.9 ± 0.9 mm versus 4.6 ± 0.9 mm, *P* < .001) and was lowest in HCM stage C (2.4 ± 0.6 mm, *P* < .001). An AAPSE <2.9 mm gave a sensitivity of 83% (95% CI 71%‐91%) and specificity of 92% (95% CI 82%‐97%) to differentiate HCM stage C from stage B. AAPSE correlated with mitral annular plane systolic excursion (*r* = .6 [.4‐.7], *P* < .001), and atrial fractional shortening (*r* = .6 [.5‐.7], *P* < .001), but showed no correlation with LV fractional shortening.

**Conclusions and Clinical Importance:**

Aortic annular plane systolic excursion is an easily acquired echocardiographic variable and might be a new measurement of LV systolic performance in cats with HCM.

**Abbreviations**
AAPSE: aortic annular plane systolic excursion
AAPSE‐D: aortic annular plane systolic excursion of dorsal aortic wall
AAPSE‐V: aortic annular plane systolic excursion of ventral aortic wall
AUC: area under the curve
CHF: congestive heart failure
CV: coefficient of variation
FW: free wall
GLS: global longitudinal strain
HCM: hypertrophic cardiomyopathy
ICC: intraclass correlation coefficient
IVS: interventricular septum
LA:Ao: left atrium‐to‐aortic root ratio
LAD: left atrial diameter
LAFS%: left atrial fractional shortening
LV: left ventricle
LVEF%: left ventricular ejection fraction
LVFS%: left ventricular fractional shortening
LVIDd: left ventricular internal dimension in end‐diastole
MAPSE: mitral annular plane systolic excursion
RPSA: right parasternal short axis view
SAM: systolic anterior motion of the mitral valve
TDI: tissue Doppler imaging

## 1 INTRODUCTION

Hypertrophic cardiomyopathy (HCM) is the most common heart disease in cats, characterized by an increased left ventricular (LV) wall thickness in the absence of other cardiovascular or systemic causes.^1^ Although primarily a disease resulting in diastolic dysfunction, declining LV systolic function in HCM and association with a worse outcome is described in humans and cats.^2^, ^3^, ^4^, ^5^, ^6^, ^7^

Systolic function of the LV is determined by a complex arrangement of myofibers which contribute to longitudinal LV shortening, circumferential and radial shortening and an axial twist.^8^ In people with HCM, reductions in LV longitudinal function, assessed by global longitudinal strain (GLS), occur before documentation of a reduced ejection fraction,^9^, ^10^ and this is documented in cats.^11^ Strain imaging requires high echocardiographic quality along with specialist software, both of which are frequently unavailable to the small animal practitioner. Some of these difficulties have been overcome by assessing mitral annular plane systolic excursion (MAPSE), which is a marker of LV longitudinal systolic function and correlates with GLS in people.^12^, ^13^

Cats with HCM with a reduced MAPSE are more likely to be in congestive heart failure (CHF) and have a worse prognosis.^2^, ^4^, ^6^ But this measurement requires a left apical view, which can be challenging to acquire especially in unstable cats. In humans, aortic annular plane systolic excursion (AAPSE) is suggested as a marker of LV longitudinal systolic function which is technically easier to obtain and correlates with MAPSE and GLS.^12^, ^14^, ^15^, ^16^

Currently there are no published reference intervals for AAPSE in cats and no reports on the diagnostic use of AAPSE in cats with HCM. We hypothesized that AAPSE would be lower in cats with HCM compared to control cats, and cats in CHF (stage C) would have the lowest AAPSE measurements. We aimed to provide AAPSE values for normal cats and determine if cats with HCM stage B have a lower AAPSE than normal control cats, and if AAPSE is associated with CHF.

## 2 MATERIALS AND METHODS

### 2.1 Animals

Multicenter retrospective case‐control study. The electronic medical records of client‐owned cats from 4 specialist referral hospitals from November 2019 to May 2022 were reviewed for cats diagnosed with HCM and normal cats. Cases were included in the study if they had a complete case record (signalment, history, physical examination and current medications), diagnostic echocardiographic examination including a right‐parasternal short‐axis view (RPSA) at the level of the aortic valve with M‐mode of sufficient quality to measure AAPSE or a RPSA of sufficient quality to perform anatomical M‐mode assessment.

Exclusion criteria for this study included systemic diseases known to affect the cardiovascular system or mimic HCM (phenocopies), such as systemic hypertension (systolic blood pressure > 180 mm Hg), hyperthyroidism (all cats >6 years of age had T4 measured), dehydration, diabetes mellitus, anemia (packed cell volume of <20%), neoplasia, and arrhythmias, congenital heart disease and cardiomyopathies other than HCM. Additionally, cases with incomplete clinical records or echocardiography examination were excluded.

Cats sedated for echocardiography with butorphanol alone or in combination with alfaxalone were not excluded, as these sedatives have been reported to have little to no effect on echocardiographic variables.^17^, ^18^

### 2.2 Echocardiographic assessment

A complete echocardiographic examination was performed in all cats including B‐mode, M‐mode and Doppler studies from standard right‐ and left‐sided views as outlined in published veterinary guidelines.^1^ If a cat had >1 echocardiogram stored over the study period, only the first echocardiogram was used. The LV wall thickness was measured at the thickest part of both the interventricular septum (IVS) and free wall (FW), in 3 2‐dimensional image planes (right parasternal long axis 4‐chamber view, right parasternal long‐axis 5‐chamber view and RPSA view at the level of the papillary muscles) using the leading‐edge to leading‐edge method, over at least 3 cardiac cycles and averaged. A diagnosis of HCM was made if LV wall thickness in any segment measured at end‐diastole on B‐mode was ≥6 mm. Normal cats were defined as maximum LV wall thickness at end‐diastole of ≤5 mm. Left atrial size was assessed by the left atrium‐to‐aortic root ratio (LA:Ao) in the RPSA view and left atrial diameter (LAD) in a right parasternal long‐axis 4‐chamber view measured at end‐systole.^19^, ^20^, ^21^ Left atrial function was assessed by calculating left atrial fractional shortening (LAFS%) from a RPSA view using M‐mode.^21^ The presence of systolic anterior motion of the mitral valve (SAM) was documented based on 2‐dimensional and color Doppler assessment of a right parasternal long‐axis 5‐chamber view, with evidence of movement of the anterior mitral valve leaflet toward the IVS in systole with turbulent flow in the LV outflow tract. The LV systolic function was measured by LV fractional shortening on M‐mode (LVFS%) from a RPSA view and where available the S′ wave of the IVS on tissue‐Doppler imaging (TDI) obtained from a left apical 4‐chamber view. Mitral annular plane systolic excursion (MAPSE) was also measured if there was sufficient quality in the left‐apical 4‐chamber view (for anatomic M‐mode) or if recorded at time of image acquisition. The LV internal dimension in end‐diastole (LVIDd) was measured from a right parasternal long‐axis 4‐chamber view using the trailing edge to leading edge method on B‐mode. The LV outflow tract velocities and velocity time integral were also recorded from a left apical 4‐chamber view.

Cats with HCM were considered as stage B if they were not receiving diuretics and did not have a history of tachypnea, dyspnea, syncope or signs of arterial thromboembolism. Cats could be included as stage B if they were on any other medication except for diuretics. Cats with HCM were in the stage C (CHF) group if they had increased respiratory rate and/or effort and either ultrasonographic or radiographic evidence of pulmonary edema or pleural effusion. Echocardiography could have been performed either before the administration of diuretics or when the cats had recently been diagnosed with CHF (within 24 hours of echocardiography).

All echocardiographic exams were performed and measured by board‐certified veterinary cardiologists or a cardiology resident under direct supervision of a board‐certified cardiologist.

### 2.3 AAPSE measurement

**FIGURE 1:**
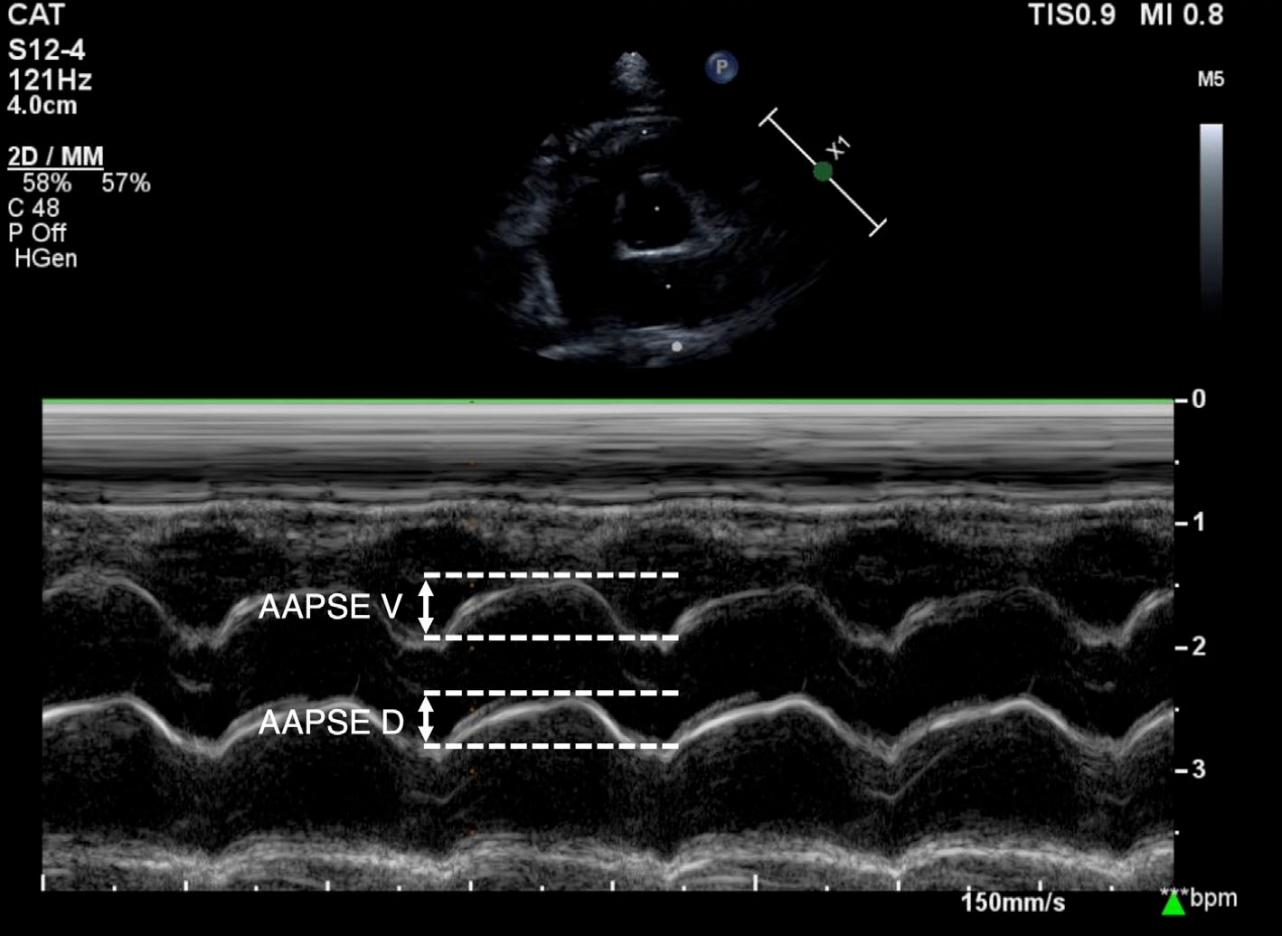
M‐mode right parasternal short‐axis view at the level of the aortic valve. To measure aortic annular plane systolic excursion (AAPSE) the cursor was placed to bisect the aorta and left atrium, with displacement of the ventral border (AAPSE‐V) and dorsal border (AAPSE‐D) measured in mm using the leading‐edge to leading‐edge method.

Aortic annular plane systolic excursion (AAPSE) was measured as described in human literature.^12^, ^22^ Both ventral and dorsal AAPSE (AAPSE‐V and AAPSE‐D respectively) were measured as shown in Figure 1 from a RPSA view at the level of the aortic valve. A minimum of 5 cardiac cycles were measured and averaged to calculate the mean values for each cat. Intra‐ and inter‐observer reliability was determined by repeat measurements of a random 10% of the cohort. Intra‐observer repeatability was determined for a single observer (Luke C. Dutton) on measurements obtained at least 1 week apart. Inter‐observer reproducibility was determined by measurements performed between 2 independent observers (Luke C. Dutton and Jose Novo Matos).

### 2.4 Statistical analysis

Statistical analysis was performed using commercially available software (GraphPad Prism version 9.3.1 for Mac OS X, GraphPad Software, San Diego, California, USA; IBM SPSS Statistics for Mac, Version 23.0. Armonk, NY: IBM Corp). Data was assessed for normality by both histogram analysis and Shapiro‐Wilk test. Parametric data are presented as mean (SD) and nonparametric data presented as median (range). Categorical data is presented as frequency and percentage. Comparison between 2 groups were carried out using Student's *t*‐test for continuous parametric data and Mann‐Whitney *U* test for continuous nonparametric data. Comparisons between 3 or more groups was performed using 2‐way ANOVA with Tukey post‐hoc analysis for continuous parametric data and Kruskal‐Wallis test for continuous nonparametric data with Dunn's post‐hoc analysis. Chi‐square test was used to compare categorical variables between 2 or more groups. Pearson's correlation coefficient was performed to assess correlation between AAPSE and echocardiographic variables of LV systolic function, forward aortic flow, LAFS% and heart rate. Strength of correlation was defined in our study as follows: 0 to .2, little or no correlation; .21 to .50, fair; .51‐.75, moderate to good; .75‐1.0 good to excellent.^23^ Receiver operating characteristic curve analysis was performed to obtain the optimal cut‐off of AAPSE to differentiate stage C from stage B cats, and the area under the curve (AUC) was calculated. The latter was defined as follows: poor, 0‐.20; slight, .21‐.40; moderate, .41‐.60; good, .61‐.80; and very good, .81‐1.0.^24^ AAPSE values for the control cohort were based on the 90% confidence intervals as previously recommended for the sample size.^25^ *P* values <.05 were considered statistically significant. Intra‐ and inter‐observer reliability and variability for AAPSE were quantified by intraclass correlation coefficient (ICC) and average percent coefficient of variation (CV), respectively. Intraclass correlation coefficient (ICC) estimates and their 95% confidence intervals were calculated based on a single‐observer, absolute agreement, 2‐way mixed‐effects model. Percent CV was calculated as (SD of the measurements/average of the measurements) × 100. In our study, an ICC > .75 and CV < 10% were considered to indicate excellent measurement reliability and low measurement variability, respectively.^26^

### 2.5 Power calculation

Since no AAPSE measurements are reported for cats, the power calculation was based on reported measurements for MAPSE, which in humans is similar to and correlated with AAPSE values.^14^ Based on this assumption a group size of n = 30 cats would provide a power of 93% to detect a >10% difference in AAPSE measurements between groups (assuming an AAPSE of 5.2 mm in normal cats), with an *α* value of .05.

## 3 RESULTS

### 3.1 Study cohort

We enrolled 175 cats, comprising 60 control cats, 61 cats with HCM stage B and 54 cats with HCM stage C (29 with pulmonary edema, 11 with pleural effusion, and 14 with both). Cohort characteristics are shown in Table 1. The median age was 7.1 years (0.4‐20.7 years). Cats with HCM stage C were older than control cats (median 9.1 years (0.6‐20.7) versus 5.5 years (0.4‐17.2), *P* = .009). Male cats were overrepresented (114/175 cats, 65%). There was no difference in sex distribution between the groups (*P* = .49). The median bodyweight was 4.5 kg (2.1‐7.5 kg). Control cats had a lower bodyweight compared to cats with HCM stage B (median 4.0 kg (2.1‐7.0 kg) versus 5.0 kg (2.8‐9.6 kg)). Domestic shorthairs were the most common breed (113/175 cats, 65%), other breeds are shown in Table 1. Most cats had a murmur on auscultation (108/175 cats, 62%), and in a minority of cats a gallop sound was detected (17/175 cats, 9.7%). The heart rate at time of evaluation was significantly higher for HCM stage C cats compared to control cats (180 bpm (140‐257 bpm) vs. 175 bpm (110‐240 bpm), *P* = .02). There was no difference in heart rate between control cats and HCM stage B cats (*P* = .21) or between HCM stage B and HCM stage C cats (*P* = .92). A total of 6 cats were receiving pimobendan at the time of the scan (all cats with HCM stage C).

**TABLE 1:** Cohort characteristics and echocardiographic values for the cats in this study.

	Control	HCM stage B	HCM stage C	*P* value
N	60	61	54	‐
Age (years)	5.5 (0.4‐17.2)^γ^ [n = 60]	6.0 (0.6‐15.6) [n = 61]	9.1 (0.6‐20.7)^α^ [n = 53]	**.009**
Post‐hoc analysis (Age): Control vs. HCM stage B: *P* = .63; control vs. HCM stage C: *P* = .007; HCM stage B vs. HCM stage C: *P* = .19				
Body weight (kg)	4.0 (2.1–7.0)^β^ [n = 56]	5.0 (2.8‐9.6)^α^ [n = 56]	4.3 (2.6‐9.0) [n = 53]	**.006**
Post‐hoc analysis (Body weight): control vs. HCM stage B: *P* = .006; control vs. HCM stage C: *P* = 1.0; HCM stage B vs. HCM stage C: *P* = .07				
Sex (M/F)	36/24	43/18	35/19	.48
Breed	36 DSH, 6 Bengal, 4 Maine Coon, 3 Sphynx, 3 Ragdoll, 7 other breeds*	42 DSH, 5 Ragdoll, 3 British Shorthair, 3 Bengal, 3 DLH, 2 Sphynx, 3 other breeds**	37 DSH, 4 DLH, 3 Persian, 2 Maine Coon, 8 other breeds***	‐
Medication (at time of echocardiography)	‐	Clopidogrel (n = 6) Rivaroxaban (n = 2)	Rivaroxaban (n = 50) Furosemide (n = 32) Clopidogrel (n = 23) Pimobendan (n = 6) Atenolol (n = 1) Benazepril/spironolactone (n = 1)	‐
Murmur (Y/N)	34/26 (57%)^β^	50/11 (82%)^αγ^	22/34 (41%)^β^	**<.001**
Post‐hoc analysis (murmur): control vs. HCM stage B: *P =* .002 control vs. HCM stage C: *P =* .06 HCM stage B vs. HCM stage C: *P* < .001				
Heart rate (bpm)	175 (110‐240)^β^	180 (120‐255)^α^	180 (140‐257)	**.02**
Post‐hoc analysis (HR): control vs. HCM stage B: *P* = .21; control vs. HCM stage C: *P* = .02; HCM stage B vs. HCM stage C: *P* = .92				
SAM (Y/N)	0/60^βγ^	39/22^αγ^	21/33^αβ^	**<.001**
Post‐hoc analysis (SAM): control vs. HCM stage B: *P* < .001; control vs. HCM stage C: *P* < .001; HCM stage B vs. HCM stage C: *P* = .01				
LVFS%	50.1 (±9.9) [n = 59]	53.5 (±13.0)^γ^ [n = 61]	46.4 (±12.6)^β^ [n = 52]	**.008**
Post‐hoc analysis (LVFS%): control vs. HCM stage B: *P* = .35; control vs. HCM stage C: *P* = .18; HCM stage B vs. HCM stage C: *P* = .006				
LA:Ao	1.4 (1.1‐1.6)^γ^ [n = 59]	1.4 (0.9‐2.4)^γ^ [n = 61]	2.1 (1.2‐3.7)^αβ^ [n = 54]	**<.001**
Post‐hoc analysis (LA:Ao): control vs. HCM stage B: *P* = .36; control vs. HCM stage C: *P* < .001; HCM stage B vs. HCM stage C: *P* < .001				
LAD (mm)	14.3 (±1.4)^βγ^ [n = 59]	17.0 (±3.5)^αγ^ [n = 61]	22.1 (±5.1)^αβ^ [n = 54]	**<.001**
Post‐hoc analysis (LAD): control vs. HCM stage B: *P* < .001; control vs. HCM stage C: *P* < .001; HCM stage B vs. HCM stage C: *P* < .001				
LAFS%	30.7 (±7.1)^βγ^ [n = 60]	27.3 (±9.5)^αγ^ [n = 61]	12.2 (±4.7)^αβ^ [n = 54]	**<.001**
Post‐hoc analysis (LAFS%): control vs. HCM stage B: *P =* .04; control vs. HCM stage C: *P* < .001; HCM stage B vs. HCM stage C: *P* < .001				
IVS TDI S′ (cm/s)	7.3 (±2.0)^γ^ [n = 25]	7.5 (±2.1)^γ^ [n = 9]	4.4 (±1.0)^αβ^ [n = 14]	**<.001**
Post‐hoc analysis (IVS TDI S′): control vs. HCM stage B: *P =* .96; control vs. HCM stage C: *P* < .001; HCM stage B vs. HCM stage C: *P* < .001				
LVOT Vmax (m/s)	1.0 (0.5‐1.5)^β^ [n = 56]	1.5 (0.7‐4.9)^αγ^ [n = 53]	1.0 (0.4‐4.7)^β^ [n = 48]	**<.001**
Post‐hoc analysis (LVOT Vmax): control vs. HCM stage B: *P* < .001; control vs. HCM stage C: *P =* .83; HCM stage B vs. HCM stage C: *P* = .006				
Aortic VTI (cm)	9.6 (±2.3)^β^ [n = 37]	18.0 (±12.2)^α^ [n = 21]	14.3 (±11.8) [n = 41]	**<.001**
Post‐hoc analysis (Aortic VTI): control vs. HCM stage B: *P =* .005; control vs. HCM stage C: *P =* .08; HCM stage B vs. HCM stage C: *P =* .32				
MAPSE‐FW (mm)	5.3 (±1.1)^βγ^ [n = 40]	4.6 (±1.2)^αγ^ [n = 28]	3.2 (±1.2)^αβ^ [n = 25]	**<.001**
Post‐hoc analysis (MAPSE‐FW): control vs. HCM stage B: *P =* .04; control vs. HCM stage C: *P* < .001; HCM stage B vs. HCM stage C: *P* < .001				
MAPSE‐IVS (mm)	5.0 (±1.1)^βγ^ [n = 39]	4.4 (±1.0)^αγ^ [n = 28]	3.0 (±0.9)^αβ^ [n = 25]	**<.001**
Post‐hoc analysis (MAPSE‐IVS): control vs. HCM stage B: *P =* .04; control vs. HCM stage C: *P* < .001; HCM stage B vs. HCM stage C: *P* < .001				
AAPSE‐V (mm)	4.6 (±0.9)^βγ^ [n = 60]	3.9 (±0.9)^αγ^ [n = 61]	2.4 (±0.6)^αβ^ [n = 54]	**<.001**
Post‐hoc analysis (AAPSE‐V): control vs. HCM stage B: *P* < .001; control vs. HCM stage C: *P* < .001; HCM stage B vs. HCM stage C: *P* < .001				
AAPSE‐D (mm)	4.2 (±0.7)^βγ^ [n = 60]	3.6 (±0.8)^αγ^ [n = 61]	2.3 (±0.7)^αβ^ [n = 54]	**<.001**
Post‐hoc analysis (AAPSE‐D): control vs. HCM stage B: *P* < .001; control vs. HCM stage C: *P* < .001; HCM stage B vs. HCM stage C: *P* < .001				

*Note*: Normally distributed data are presented as mean (±SD), non‐normally distributed data is presented as median (range). Categorical data are presented as frequency and percentage. Greek values indicate statistically significant result between groups based on post‐hoc analysis. *α* = compared to control group, *β* = compared to hypertrophic cardiomyopathy (HCM) stage B group, *γ* = compared to HCM stage C group. Bold values indicate *P* < .05.

Abbreviations: AAPSE, aortic annular plane systolic excursion; AAPSE‐V, aortic annular plane systolic excursion from the ventral aortic wall; AAPSE‐D, aortic annular plane systolic excursion from the dorsal aortic wall; LAAo, left atrium to aortic root ratio; LAD, left atrial diameter; LAFS%, left atrial fractional shortening; LVOT Vmax, left ventricular outflow tract maximum flow velocity; LVFS%, left ventricular fractional shortening; M/F, male or female; MAPSE, mitral annular plane systolic excursion; MAPSE‐FW, mitral annular plane systolic excursion from the free wall; MAPSE‐IVS, mitral annular plane systolic excursion from the interventricular septum; Other breeds: * 1 cat of each breed: Russian Blue, Persian, Exotic Shorthair, British Longhair, Norwegian Forest, Birman, Thai; ** 1 cat of each breed: British Longhair, Exotic Shorthair, Chartreux; *** 1 cat of each breed: Ragdoll, British shorthair, Himalayan, Norwegian Forrest, Chartreux, Sphynx, British Longhair, Cornish Rex. SAM, systolic anterior motion of the mitral valve; IVS TDI S′, systolic velocity of the interventricular septum obtained from tissue Doppler imaging; VTI, velocity time integral; Y/N, yes or no.

### 3.2 Echocardiographic data

Echocardiographic data is summarized in Table 1. A total of 12 cats were sedated for echocardiography, either using butorphanol alone (n = 9) or a combination of butorphanol and alfaxalone (n = 3). Within the HCM stage B group, 48/61 (79%) cats had an LA:Ao ≤1.6 (1.33 mm ± .15) and LAD ≤16 mm (15.6 mm, ±1.7) and 13/61 (21%) cats had an LA:Ao >1.6 (1.92 ± .25) and LAD >16 mm (21.5 mm, ±4.5).

Cats with HCM stage C had a lower LVFS% compared to stage B cats (*P* = .001) but there was no difference between control cats and stage C cats (*P* = .13) or between control cats and stage B cats (*P* = .46). The LA:Ao was larger in cats with HCM stage C compared to stage B and control cats (*P* < .001). The LAD was larger in cats with HCM stage B compared to control cats (*P* < .001) and larger in stage C compared to stage B and control cats (*P* < .001). The LAFS% was lower in cats with HCM stage B and C compared to control cats and between cats with HCM stage C and B (*P* < .001). The IVS TDI S′ was lower in cats with HCM stage C compared to stage B and controls (*P* < .001). Cats with SAM had higher LV outflow tract velocities compared to cats without SAM (3.10 m/s ± 1.96, n = 61 vs. 0.93 m/s ± 0.23, *P* < .001, n = 103). More cats with HCM stage B had SAM compared to stage C cats (*P* = .01). Both MAPSE IVS and MAPSE FW were lower in HCM stage C compared to stage B cats, in which values were lower than for control cats (*P* < .001).

### 3.3 Aortic annular plane systolic excursion values in control cats and reliability

We obtained AAPSE values for cats without evidence of HCM using our control cohort (Table 2). Normal cats had an AAPSE‐V of 4.6 mm (90% CI 4.2‐4.8 mm) and an AAPSE‐D of 4.2 mm (90% CI 3.3‐5.1 mm). AAPSE showed excellent intra‐ and inter‐observer reliability (ICC = 0.98 (95% CI .96‐.99) and 0.76 (95% CI .69‐.84), respectively).

**TABLE 2:** Values for aortic annular plane systolic excursion (AAPSE) based on measurements from the control group of cats (n = 60).

	AAPSE ventral	AAPSE dorsal
Mean (mm)	4.6	4.2
SD	0.85	0.73
90% confidence interval	4.2‐4.8	3.3‐5.1
Range	3.3‐6.8	2.6‐6.1

### 3.4 Aortic annular plane systolic excursion in HCM compared to control cats

**FIGURE 2:**
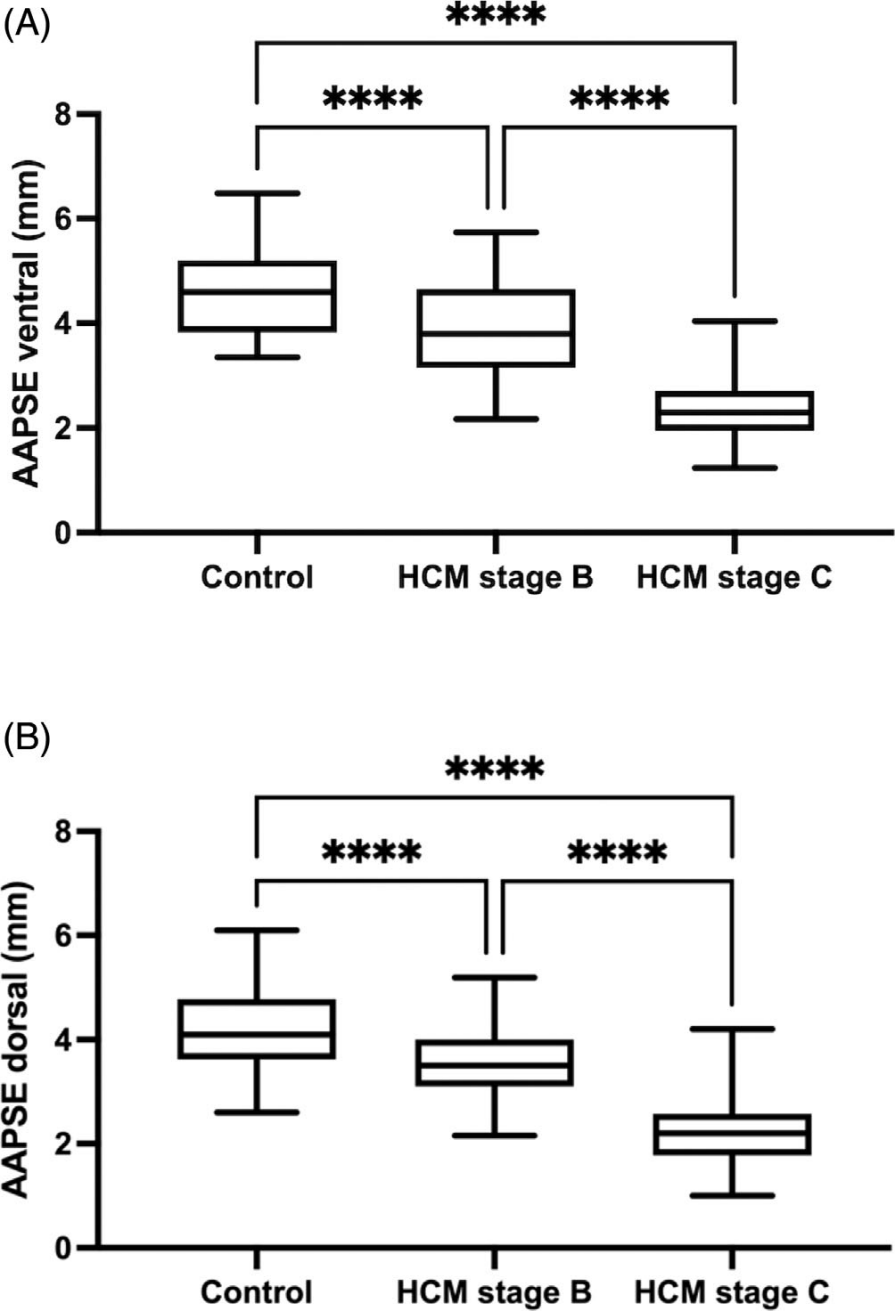
Box and whisker plots showing distribution of aortic annular plane systolic excursion (AAPSE) measurements for each of control cats, cats with hypertrophic cardiomyopathy (HCM) stage B and stage C. AAPSE ventral was lower in cats with HCM stage B and lowest in stage C cats (A, *P* < .001). Distribution of AAPSE dorsal measurements for each group show a similar pattern (B, *P* < .001). ****P < .001 based on ANOVA with post‐hoc Tukey analysis.

**FIGURE 3:**
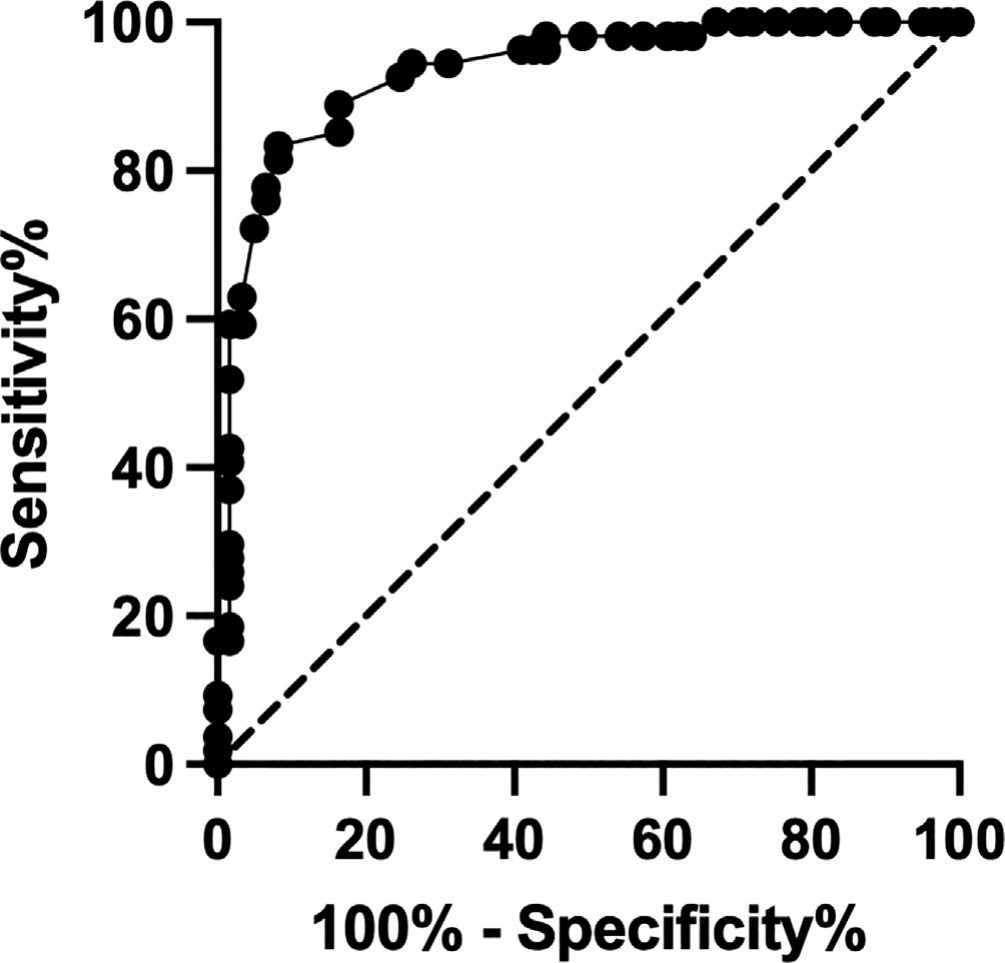
Receiver operating characteristic (ROC) curve to assess the ability of aortic annular plane systolic excursion (AAPSE) from the ventral aortic wall (AAPSE‐V) to differentiate cats with hypertrophic cardiomyopathy (HCM) stage B and stage C cats. The AUC was 0.93. An AAPSE‐V cut‐off of 2.9 mm gave a sensitivity of 83% and specificity of 92% to differentiate HCM stage C from stage B cats.

Compared to control cats, both AAPSE‐V and AAPSE‐D were lower in cats with HCM stage B (*P* < .001, Table 1 and Figure 2A,B), and cats with HCM stage C had both an AAPSE‐V and AAPSE‐D that was lower than cats with HCM stage B (*P* < .001, Table 2 and Figure 2A,B). In addition, receiver operating characteristic curves were obtained for both AAPSE‐V and AAPSE‐D to differentiate cats with HCM stage C from stage B. The AAPSE‐V receiver operating characteristic curve had a similar AUC compared to AAPSE‐D, and both were good at predicting stage C compared to stage B cats (0.93, 95% CI 0.89‐0.98 versus 0.90, 95% CI 0.84‐0.96, Figure 3). An AAPSE‐V cut‐off of 2.9 mm gave a sensitivity of 83% (95% CI 71%‐91%) and specificity of 92% (95% CI 82%‐97%) to differentiate cats with HCM stage C from stage B. Similarly, an AAPSE‐D cut‐off of 2.9 mm gave a sensitivity of 80% (95% CI 67%‐88%) and specificity of 82% (95% CI 71%‐90%) to differentiate cats with HCM stage C from stage B.

### 3.5 Correlation of AAPSE with other echocardiographic variables

**FIGURE 4:**
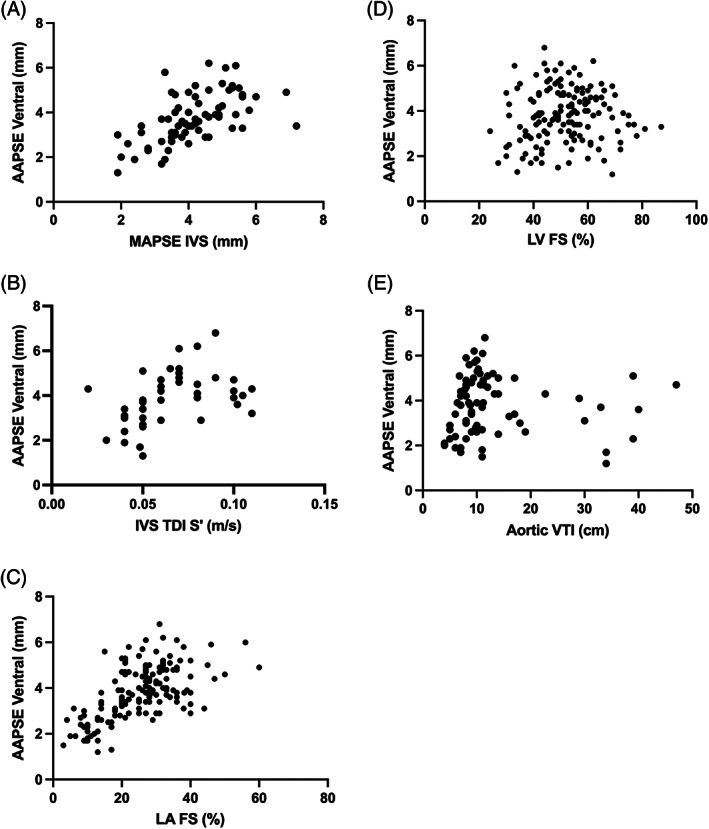
Correlation of aortic annular plane systolic excursion (AAPSE) with other echocardiographic indices. Aortic annular plane systolic excursion from the ventral aortic wall (AAPSE‐V) showed a positive correlation with mitral annular plane systolic excursion (MAPSE) from the interventricular septum (IVS; A), tissue Doppler systolic velocity of the interventricular septum (IVS TDI S′; B) and left atrial fractional shortening (LAFS%; C). There was no correlation with left ventricular fractional shortening (LVFS%; D). There was also no correlation between AAPSE and aortic velocity time integral (VTI; E).

Aortic annular plane systolic excursion of ventral aortic wall and AAPSE‐D showed a moderate positive correlation with MAPSE IVS (*r* = .6 [.4‐.7] for both, *r* ^2^ = .36, *P* < .001, Figure 4A) and LAFS% (*r* = .6 [.5‐.7] for both, *r* ^2^ = .36, *P* < .001, Figure 4C), and a fair positive correlation with IVS TDI S′ (*r* = .5 (.2‐.7), *r* ^2^ = .25, for AAPSE‐V and *r* = .42 (.15‐.64), *r* ^2^ = .18, for AAPSE‐D, *P* = .002 and *P* = .003 respectively, Figure 4B). There was no correlation with LVFS% (*r* = .1 [−.06‐.25], *r* ^2^ = .01, *P* = .23, Figure 4D), aortic velocity time integral (*r* = −.06 [−.28‐.16], *r* ^2^ = 0.004, *P* = .60, Figure 4E) and peak transaortic flow velocity (*r* = −.10 [−.26‐.06], *r* ^2^ = .01, *P* = .23). There was also no correlation between AAPSE and heart rate (*r* = −.02 [−.18‐.14], *r* ^2^ = .0004, *P* = .79). When cats with SAM were removed from analysis, there was a fair positive correlation between AAPSE and aortic velocity time integral (*r* = .4 [.19‐.60], *r* ^2^ = .16, *P* < .001, n = 65) and LV outflow tract velocity (*r* = .32 [.13‐.49], *r* ^2^ = .1, *P* < .001; n = 103).

## 4 DISCUSSION

In this study, we aimed to assess AAPSE in a cohort of cats without structural heart disease (control group) and those with HCM stages B and C. We demonstrated that AAPSE is lower in cats with HCM compared to control cats and is lowest in cats with HCM stage C. We provided AAPSE values for normal cats. AAPSE demonstrated a high sensitivity and specificity to distinguish cats with HCM stage B from stage C. AAPSE showed positive correlation with other indices of LV longitudinal systolic function (MAPSE, TDI S′), but seemed independent of indices of short axis function (ie, LVFS%) and had a weak association with aortic flow variables. Furthermore, AAPSE showed excellent intra‐ and inter‐observer repeatability and reproducibility, respectively.

Although HCM is typically defined as a disease resulting in diastolic dysfunction, it is well established both in cats and humans that as the disease progresses, systolic function is also impaired.^2^, ^27^, ^28^, ^29^ In addition, although HCM is typically associated with increased (hyperdynamic) LVFS%, this variable more likely represents changes in loading conditions and LV internal dimensions due to hypertrophy, rather than a true increase in systolic function.^28^, ^30^, ^31^, ^32^ Cardiac contraction depends on longitudinal shortening, circumferential shortening, radial shortening and an axial twist.^8^, ^33^ Assessment by echocardiography using LVFS% measures short axis systolic function, LV ejection fraction assess both longitudinal and circumferential systolic function and global longitudinal strain and MAPSE measure longitudinal systolic function.^34^, ^35^, ^36^ In humans, GLS is more sensitive compared to LV ejection fraction to detect declining systolic function and is a better predictor of adverse outcome in a variety of cardiac diseases.^7^, ^9^, ^10^, ^36^, ^37^, ^38^ In people with HCM both GLS and MAPSE reduce as the disease progresses.^3^, ^7^, ^10^, ^38^ Therefore, in humans GLS and MAPSE are used to document systolic dysfunction in patients with HCM and this correlates with disease severity and acts as a prognostic indicator. Similarly in cats, reduction in MAPSE is associated with a worse prognosis^6^ and cats with HCM have a reduced GLS, suggesting reduced overall longitudinal systolic function.^11^, ^28^

In humans, displacement of the aortic root is used to assess longitudinal systolic function. This is referred to as systolic aortic root motion (SARM), aortic root systolic excursion (ARSE) and anterior aortic plane systolic excursion (AAPSE).^12^, ^14^, ^16^, ^39^ We opted to use the abbreviation AAPSE (and change anterior aortic to aortic annular) to complement MAPSE and tricuspid annular plane systolic excursion to aid ease of use and understanding. In humans AAPSE correlates with other measures of longitudinal systolic function such as MAPSE and GLS.^12^, ^15^ Although GLS was not performed in our retrospective cases, AAPSE did correlate with MAPSE and tissue Doppler S′ wave from the IVS. AAPSE has advantages over other methods to assess systolic function as it uses a standard echocardiographic view that is routinely obtained in feline echocardiography, even in unstable cats (unlike MAPSE), and requires no calculation to compare to reference values. To highlight this, AAPSE is used in people as a measure of LV systolic function in the emergency setting.^22^ Our study suggests that AAPSE could be used as a rapidly acquired and easy to learn measurement to assess LV systolic performance in the acute setting. Many emergency and critical care clinicals will be familiar with obtaining a view to assess the LA:Ao, and since AAPSE utilizes the same view, it might be feasible to a less experienced operator. However, we have not assessed this use in the present report.

Movement of the aortic root can be affected by several factors given the anatomical arrangement. The aortic root is connected to the cardiac skeleton, meaning movements of the heart base would subsequently move the aortic root. In people, the aortic root motion correlates with LV stroke volume,^16^, ^40^, ^41^ and decreases when stroke volume is diminished by higher cardiac pacing rates.^40^ In our study, there was a fair positive correlation between AAPSE and aortic velocity time integral and LV outflow tract velocity in cats without SAM, but no correlation between AAPSE and heart rate. Cats with HCM stage C had higher heart rates compared to control cats, and thus lower stroke volumes in stage C cats might have contributed to the lower AAPSE values. Since movement of the base of the heart relative to the apex is dependent on longitudinal systolic function, it seems likely that AAPSE at least in part assesses longitudinal systolic function. In addition, the aorta shares its dorsal border with the left atrium, therefore changes in the emptying and filling of the left atrium can be reflected in the movement of the aortic root.^42^ Displacement of the posterior wall of the aorta correlates with left atrial volume changes.^43^ In our study AAPSE had a moderate positive correlation with LAFS%, which could support this hypothesis. As indices of cardiac performance generally decrease with advancing disease and onset of CHF the fact that 2 measures are correlated does not necessarily imply they are assessing the same variable. It seems plausible that AAPSE is affected both by LV systolic function and left atrial filling and emptying, but data in people suggests that aortic root motion is mainly attributed to LV systolic function.^16^ Regardless of the different potential factors affecting AAPSE, this could be a useful and easy to acquire measure in cats with HCM, however the prognostic significance of AAPSE is yet to be determined.

Our study has several limitations. This was a retrospective study, therefore no standard echocardiographic protocol was followed, and thus, not all variables were assessed in every cat (eg, TDI and MAPSE). Most cats with HCM stage B had normal left atrial size (stage B1), and thus mild HCM. This might have been the cause for the overlap in the AAPSE values between control cats and HCM stage B cats. The difference between control (normal) and HCM stage B2 cats would be larger with less overlap (given the correlation between AAPSE and left atrial size). However, we do not believe this affects our ability to say that cats with HCM overall have a lower AAPSE compared to control cats. We used a referral cohort, therefore a selection bias was introduced. Additionally, we excluded cases with comorbidities that mimic HCM (HCM phenocopies) or could possibly affect AAPSE measurement. These limitations can potentially lead to an inflated performance of the echocardiographic variables used and to spectrum bias and limited challenge bias that might lead to an overestimation of the sensitivity and specificity of AAPSE.^44^, ^45^ Moreover, our results might not be applicable to a general cat population.^45^ Tissue Doppler imaging was only available for the IVS and not the FW, limiting correlation with MAPSE FW. Although AAPSE was lowest in cats with HCM stage C and correlates with other prognostic markers, further longitudinal data is required to fully assess the prognostic significance of AAPSE, ideally using multivariable analysis to assess if AAPSE is an independent outcome predictor. We did not assess observer repeatability and reproducibility for all echocardiographic measurements, but these were considered to be good to excellent given the level of experience in feline cardiology of the observers and standardization of echocardiographic views. We also did not assess the impact of different echocardiographic machines used in this study on the measurements obtained. However, we used standard B‐mode and M‐mode linear measurements that are unlikely to vary between echocardiography systems.

We did not have enough data to assess if any medications received affected AAPSE, such as furosemide or pimobendan, which could have had an effect on LV preload and left atrial and LV systolic function, respectively, and thus an impact on AAPSE values.^46^, ^47^, ^48^

In conclusion, we found that AAPSE was lower in cats with HCM, and cats with HCM stage C have the lowest values of AAPSE. This might be a new measurement of LV systolic performance, most likely related to longitudinally systolic function. We provide values for AAPSE in normal cats, and show that the measurement is easy to acquire and has excellent intra‐ and inter‐observer reliability.

## CONFLICT OF INTEREST DECLARATION

Jose Novo Matos serves as Associate Editor for the Journal of Veterinary Internal Medicine. He was not involved in review of this manuscript. No other authors declare a conflict of interest.

## OFF‐LABEL ANTIMICROBIAL DECLARATION

The authors declare no off‐label use of antimicrobials.

## INSTITUTIONAL ANIMAL CARE AND USE COMMITTEE (IACUC) OR OTHER APPROVAL DECLARATION

Approved by the Animal Ethics and Welfare Committee of the Department of Veterinary Medicine, University of Cambridge (CR704).

## HUMAN ETHICS APPROVAL DECLARATION

The authors declare human ethics approval was not needed for this study.
